# The Heart of the Matter: Health Status of Aged Care Clients Receiving Home- and Community-Based Care

**DOI:** 10.4061/2010/275303

**Published:** 2010-07-12

**Authors:** Deborah Yarmo-Roberts, Rosanne Laura Freak-Poli, Brad Cooper, Tim Noonan, Just Stolewinder, Christopher M. Reid

**Affiliations:** ^1^Department of Epidemiology and Preventive Medicine, Monash University, Victoria 3004, Australia; ^2^Baptcare, Eastern Metropolitan Community Packages, Victoria 3134, Australia

## Abstract

*Objective.* To determine the current health status of home based elderly clients receiving government funded aged care packages. *Design.* Prospective Observational study. *Setting.* Community based, home care program in Australia. *Participants.* Community-dwelling older adults receiving aged care packages. *Measurements.* A comprehensive test battery of physical, mental and social scales were completed including a Caregiver Strain Index where appropriate. *Results.* 37% of the 334 subjects were male and the mean age was 81 ± 8 years. Physical functioning was low compared to the Australian population. Depression was highly prevalent with 15.9% severely depressed and 38.7% mildly depressed. 26% of clients screened positive for dementia. Relatively good levels of social support were reported, however social networking activity levels were low. Sixty one percent of clients had caregivers, of whom 63.3% had high levels of strain. Strain was higher in caregivers of clients on higher levels of care (78.5% versus 50.6% highly strained). *Conclusion.* The data suggests that as a group there is a high degree of comorbidity, and depression, dementia and caregiver strain are highly prevalent. The findings may aid administrators and health policy planners in directing resources to key areas impacting on health outcomes in this group.

## 1. Introduction

The aged population is expanding and many countries are facing one quarter of their population being over aged 65 in the next 20–30 years [1, 2]. A global ageing society has encouraged stakeholders to think resourcefully about the changing needs of the elderly including the provision of home and community-based services. Many initiatives in provision of home and community based services have been put into practice with common aims to improve the functional, emotional and/or social support of clients or at least slow down the rate of declining health. One of these initiatives in Australia is the provision of “community-aged care packages” which are a government funded range of services that are organised for a client depending on individual need so that the client can remain in their own home as opposed to moving into residential low or high level care.

The measurement of the impact of community-aged care package provision on client health status is essential in any evaluation of their effectiveness and efficiency. There is evidence in the literature showing factors related to demographics, physical functioning, emotional wellbeing, social support, and caregiver strain can influence the requirement for individuals requiring higher levels of care [3–8] and moving away from home. However, whilst it is assumed that aged care packages enhance or maintain the health of clients, there is a lack of data on the health status of clients receiving community-aged care packages which is likely to be fundamental component to their success.

The information that is available through public documents focuses on service usage and service approach rather than health status of clients and caregivers [9, 10]. The AdHOC study in Europe is one of the few exceptions which used a standardised assessment instrument measuring socio-demographic, physical, and cognitive characteristics across 11 European countries and found significant differences across regions [11].

### 1.1. Aged Care Packages in Australia

The Community-Aged Care Packages (CACP), Extended Care at Home Packages (EACH), and Extended Care at Home Dementia (EACH Dementia) aged care packages are an Australia wide program funded, regulated and monitored by the Australian Commonwealth Government [12]. The aged care packages are targeted at frail older people living in the community who require assistance that would otherwise necessitate them moving into assisted living (low level residential care) or nursing home care (high level residential care). CACP packages are individually designed and coordinated for each client and are based on the client's particular needs [12]. The types of services may include assistance with bathing, meal preparation, laundry, dressing, transport, housework, temporary in-home respite, home maintenance or social activities or pastoral care.

EACH and EACH Dementia packages are the same type of organized services but often include the additional assistance of registered nursing care and care by an allied health professional. To be eligible to receive either of these packages, the client needs are assessed by a clinical team (referred to as an Aged Care Assessment Team) to determine the required care level. The eligibility assessment is also part of an Australia wide program. The team is trained to assist clients in determining the kind of care that will best meet their needs when they are no longer able to manage at home without assistance [13].

Across Australia in 2006, there were 1,011 identified service outlets providing CACP packages. In contrast, only 157 service outlets nationally operated EACH packages reflecting the more recent commencement of the EACH program relative to the CACP program [13]. There are also a growing number of private providers that offer nursing and home assistance. In Victoria (one of the states within Australia), there were 9116 CACP packages (31803 Australia wide) and 720 EACH packages (2131 Australia wide) provided in 2006 [14]. Other types of home care services include a federally funded program called Home and Community Care (HACC), providing direct service provision without case management (or a coordinated package of care) to clients with basic needs. This level of care is broad based with limited tailoring to individual client needs. The Australian Government expects people to transition from HACC to more intense and personalized levels of support via CACP and EACH packages [13].

The packages are not targeted at “the disabled” with eligibility based on the prevalence of aged related health conditions. The issue is compounded because disability services are funded by the State Government and Aged Care Packages funded by the Commonwealth. In effect the Aged Care system does not see “the disabled” until they reach the age of 65 when they transition from being a person with a disability to a person with aged-related issues (associated also with a disability).

### 1.2. The PITCH Study

The primary objective of the Predictors Influencing The Change in Health Status of the Elderly in Community Care (PITCH) study was to examine the current health status for BC clients on Community-Aged Care Packages (CACP), Extended Aged Care at Home (EACH) and Extended Aged Care at Home for the Dementia (EACH Dementia) packages of care. The level of caregiver strain associated with caring for clients was also a key objective. (In the context of this study the “caregiver” refered to the informal unpaid people that contribute to a persons' care—often a spouse or family member. The caregiver was selected at the same time as the participant.

## 2. Methods

The PITCH Study is a prospective longitudinal observational study of Victorian clients on CACP, EACH, and EACH Dementia aged care packages provided by a major care package provider, Baptcare Pty Ltd (BC). Permission to conduct the study was gained from the Monash University Human Ethics Committee. 

### 2.1. Participants

Study participants inclusion criteria were as follows:

- men and women who have an aged related illness/condition as determined by the Aged Care Assessment Team,
- eligible to receive either a CACP, EACH, or EACH dementia package,
- willing and able to provide informed consent.

The study was conducted across the state of Victoria, Australia. Participants resided amongst large cities (Melbourne), regional centres, small towns, and rural communities. Participants were already enrolled in care packages prior to the study, representing the natural communities in which they reside.

If the client was unable to complete the questionnaire themselves, caregivers were asked to complete a shorter questionnaire as an informant for the client.

### 2.2. Measurements

Demographics and medical history were collected through the use of a client profile Questionnaire and medical record review.

Surveys were selected based on the following criteria; relevant for an aging population, relevant for someone not at optimal health, self-reported administration, easy to understand and administer, short in length. Although this study does not specify “agreed-upon” instruments, it allows the variability between instruments to be identified. 

The health surveys were chosen to represent four overarching health themes: physical functioning, emotional wellbeing (depression, cognitive decline, emotional representations of ageing), social support (or community connectedness), caregiver strain). All scales are scored 0 to 100 unless otherwise indicated.

The SF-36 [14] was used as a measure of quality of life and includes two summary measures: physical functioning and mental health. Low scores indicate a limitation in performing physical activities or mental health and scores are comparative to the average Australian population score of 50.

The Independent Activities of Daily Living (IADL) instrument was used to assess the functional ability and canvases issues such as using the telephone, doing housekeeping and laundry, and managing finances [15]. A higher score indicates a higher functional ability. 

The 15-question Geriatric Depression Scale (GDS) was used in this study [16, 17]. Higher scores represent higher levels of depression and scores can be categorized as: normal = 0–4; mild depression = 5–9; severe depression = 10–15. The Modified Mini-mental Scale (3MS) is a brief screening test for cognitive function. Scores below 77 (out of 100) are an indication of impaired cognitive function [18, 19]. The Aging Perceptions Questionnaire (APQ) was developed to assess individuals' perceptions of the impact of their own ageing [20], with subscales representing different aspects.

The abbreviated (11 item) Duke Social Support Index (DSSI) was used in this study as it measures social support and the use of health related services across four dimensions: social networks, social interaction, subjective support, and instrumental support. Higher scores indicate lower levels of loneliness and higher levels of support [21]. 

The Social Network Scale (SNS) assesses the use of health services and lifestyle, and was originally developed in Australia to measure social isolation and loneliness in ageing populations [22]. The SNS has two subscales: “utilization of services” lists different health services, and the “utilization of activities” scores the involvement in a wide range of activities such as gardening, shopping, babysitting, sport/dance, work, and going to a church or a club. 

The Carer Strain Index (CSI) measures caregiver strain in relation to the care they are providing to the client. Higher CSI scores indicate a greater level of strain, and scores above 53.8 (out of 100) indicate greater levels of strain [23]. 

Statistical analyses were undertaken on all scales to test if there were differences between the different package types using independent *t-*tests, Chi-square or Fisher exact procedures where appropriate. Results are reported as means ± standard deviations for continuous variables and frequencies and percentages for categorical variables. Analyses were conducted using either SPSS (release 15.0.0 for Windows, SPSS Inc) or STATA (version 9.2, StataCorp LP, College Station, TX) statistical software.

## 3. Results

Of the 550 BC clientele identified to participate in the study, 73 were no longer eligible due to death or relocation and 334 attended a baseline visit (69.4%). 70% of the cohort was receiving CACP, 28% receiving EACH and the remainder receiving EACH dementia packages. Due to this small group, EACH Dementia data were included in those receiving EACH packages. 

**Figure 1:**
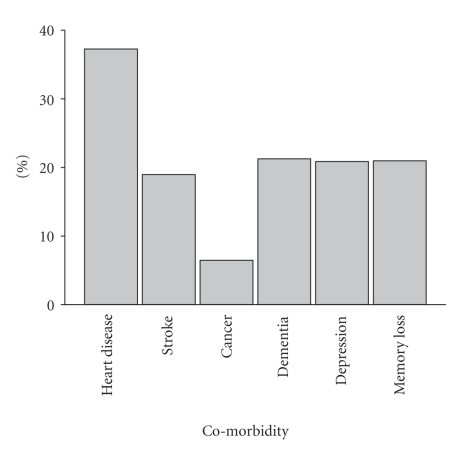
Prevalence of comorbid conditions in the PITCH population.

37% of participants were male and the mean age was 81 ± 8 years. 61% had carers. As a group, 95% were Caucasian, 70% born in Australia and had been receiving BC packages for 1.0 ± 0.6 years. 30% were from rural locations. Figure 1 illustrates the prevalence of comorbid conditions in the PITCH population. Heart disease was the major comorbid condition with high levels of depression and dementia being reported.

## 4. Health Status

The baseline health status characteristics of the PITCH cohort are shown in Table 1. Recipients of EACH packages were less physically able (significantly lower SF36 physical component and IADL scores) than those receiving CACP packages. In addition, significant increased feelings of depression were reported in EACH recipients however there was no significant difference in cognitive function levels between the groups. There were no differences in health measures between rural or metropolitan CACP or EACH recipients.

### 4.1. Social and Community Service Interaction

At baseline, the mean social support index (DSSI) score was 79.63 indicating a relatively good level of social support overall with 75% of clients scoring 70 or higher. There were no significant differences between package type or locations in relation to social support. The SNS utilisation scores appeared relatively low with a mean of 26 (scored 0–100) and there were no statistically significant differences between package types. Whilst for both service and social interaction was slightly higher in metropolitan locations, the differences were not statistically significant.

A summary of positive and negative perceptions of the consequences of ageing are shown in Table 2. There were no statistically significant difference for APQ scores between CACPS and EACH clients with total scores of 60.9 ± 7.0 and 60.3 ± 7.1, respectively, *P* = .58.

### 4.2. Carer Strain

61% of participants reported having a carer of which 90% were either, wife, husband or daughter. The mean score overall was 57.6, and scores above 53.8 indicates a high level of carer strain. At baseline 114 carers (63%) had levels of carer strain above 53.8. Carers of EACH recipients reported significantly higher levels of carer strain than CACP (mean scores of 66.0 ± 22.7 versus 49.6 ± 27.4, resp.). This was particularly the case in metropolitan areas, with metro EACH carers averaging 25.5% more strain than metro CACP carers.

## 5. Discussion

To the best of our knowledge, this is one of the first accounts of health status amongst care package recipients and their caregivers in Australia. Of all measures of health status, the levels of depression, established cardiovascular disease, dementia, and caregiver strain were identified as the major health concerns. These data are essential to provide an understanding of the health status of the client population on aged care packages and the implications of health status on the major objective of care package provision—keeping people at home rather than in higher dependency settings.

The age and gender characteristics of this population are similar to the national figures for clients receiving CAPC and EACH participants across the country (63% and 49% being over the age of 80 years, resp.) [24]. 

The instruments chosen in this study were selected on the basis of widespread use and validated in clinical and research programs for elderly people. The breadth of instruments captured the physical, emotional, and social health related status of clients, which is endorsed in the definition of health used by the World Health Organisation [25]. The utility of various survey questionnaires to measure the health status of clients receiving community-aged care packages is largely unknown. Although there are many validated studies from which to choose for the elderly population, there is not uniform agreement on the suite of tools which are deemed the most effective in measuring clients' functional, emotional, and social health.

By providing a number of assessment tools that measured (to some degree) similar outcomes, variability between measures were identified. For example, the SF-36 was useful at identifying the low level of physical functioning among participants generally, however, variability between BC packages were limited. Functional ability variability between BC packages was better assessed by the IADL. The GDS, SNS, and CSI questionnaires were also useful at determining differences between BC packages. The 3MS may be useful to assess those CAPC clients likely to progress the more advanced care requirements such as EACH Dementia clients as only 0.87% of the 77 participants identified with cognitive impairment were on the EACH Dementia package. 

In comparison to a Finish study [26] (average age 81.2 ± 4.6 years, 74% female), BC clients have lower functioning ability. This Finish study found that those without dementia had an average IADL score of 85.00 ± 20.00, while those with dementia averaged 45.00 ± 31.25. These results illustrate that BC clients' ability to perform common tasks appears to be lower. This is confirmed by the findings for the SF-36 on physical function indicating that the BC client group has higher needs in relation to assistance with physical tasks than the average population.

The rates of depression found in this study were not dissimilar to other studies of comparable community-dwelling older populations in Australia [27] (average age 86.3 ± 6.9 years, 70.6% female) which found that 53.3% of the group met the screening criteria for depression defined by the GDS. This reinforces the concern that this group can benefit from interventions which reduce depression. 

BC clients had a higher proportion of 3MS-screened dementia compared to a study looking at 8,697 community-dwelling Canadians [28] (average age 75 ± 7.1 years, 59.3% females) who had higher levels of cognitive ability. Of the community-dwelling Canadians only 5.5% screened positive for dementia by the 3MS, which was significantly less than BC clients. 

The level of social support, as measured by the DSSI, indicated that BC clients had varying levels of social support. In comparison to an Australian study [29] with women aged 70–75 (average DSSI score 85.33 ± 9.67) BC clients had lower social support. Accessibility to health services and related assistance varies between metropolitan cities and rural settings and it may be hypothesized that social support of clients would be different due to location and access to services. Hence, the finding of similar outcomes between metropolitan and rural is of interest.

Identifying individuals with key health issues at the commencement of the BC program may direct resources to better manage these conditions with the objective of maintaining the health of these individuals over the long term.

On average, BC clients perceived ageing has both positive and negative consequences and that they have control over positive outcomes associated with aging. BC clients did not perceive they had as much control over negative experiences or outcomes associated with ageing and that anxiety, depression, fear and anger were present. When compared to older Irish adults [20] (average age 74.1 + 6.8 SD years, 57% female) the BC clients had similar perceptions about positive consequences and positive control in regards to aging. However, BC clients believed that they had much less control over negative experiences associated with aging and that there were more negative consequences and more negative emotional responses associated with aging. These results suggest that there are opportunities for those administering care packages to play a particularly important role in helping clients develop a more positive response to some of the negative consequences of ageing. 

Caregivers to BC clients have high levels of strain, especially in comparison to caregivers of stroke patients [30] (average score of 33.1) or compared to caregivers of cancer patients [31] (average score of 30.8). A high percentage (63%) of caregivers are classified as highly strained, especially when compared to 37% of caregivers with high levels of strain in another community-based study [32] (*P* = .043) or 39.7% of caregivers of stroke patients [33] (*P* < .001). These results show that more BC caregivers are highly strained than other unpaid caregivers [32, 33] and this percentage (63%) illustrates the necessity to target the needs of the caregiver as well as the client. The finding that EACH clients have more strained caregivers than CACP caregivers shows that the needs of caregivers may increase as the needs of the client increase.

Limitations of this study are acknowledged particularly in that the findings may not reflect heath status of care package recipients receiving support from other providers. In addition, comparison of other populations are difficult due to potential subject selection biases.

## 6. Conclusions

BC clients have lower physical function than the general Australian population, depression and dementia are highly prevalent and caregiver strain is also evident. This study has provided a level of understanding of a community-aged care population group that had not been evident to date. Further analysis of health status over time (6 month and 12 month) will assist in determining the change in health status and identify predictors (risk factors) associated with movement between packages of care. This will provide a more comprehensive understanding of this population group and will be a foundation for future evaluations seeking to determine the value of service delivery approaches.

##  Financial Disclosure

The funding for this project came from Baptcare.

##  Author Contributions

D. Roberts: concept and design, interpretation of data, preparation of manuscript. R. Freak-Poli: interpretation of data and write up of results. B. Cooper & T. Noonan: study concept and design. C. Reid: study concept and design and preparation of manuscript. J. Stoelwinder: study concept and design, preparation of manuscript.

## Figures and Tables

**Table 1:** Baseline health characteristics of the PITCH population.

Health measure	CACP	EACH	*P*-value
SF 36—Physical	32.6 ± 9.8	26.2 ± 9.3	.0001
—Mental	50.5 ± 11.2	48.8 ± 12.6	.38
IADL Score	72.5 ± 19.2	42.2 ± 22.2	.000
GDS	4.6 ± 3.3	7.8 ± 3.8	.000
3MS	81.6 ± 12.9	78.4 ± 17.6	.17

**Table 2:** Summary of APQ Subscale Results.

Subscale	PITCH Mean (sd)	Older Irish Adults (20) Mean (sd)
Consequences Positive		

(higher scores indicate strong belief that ageing has positive consequences)	72.95 (16)	74.00 (12)

Consequences Negative		

(higher scores indicate strong belief that ageing has negative consequences)	75.20 (13)	68.00 (15)

Control Positive		

(higher scores indicates more perceived control over positive experiences/outcomes associated with ageing)	75.57 (12)	76.00 (11)

Control Negative		

(higher scores indicates more perceived control over negative experiences/outcomes associated with ageing)	31.38 (15)	53.00 (14)

Emotional Representations		

(higher scores indicate negative emotional responses to ageing including anxiety, depression, fear and anger)	50.77 (18)	48.00 (15)
